# Andes virus from the 2026 *MV Hondius* outbreak does not show enhanced entry into human cells

**DOI:** 10.1080/22221751.2026.2732719

**Published:** 2026-09-10

**Authors:** Marc Carrascosa-Sàez, Rodrigo Arce, Jérémy Dufloo, Rafael Sanjuán

**Affiliations:** Institute for Integrative Systems Biology (I^2^SysBio), University of Valencia – CSIC, Paterna, Spain

The recent outbreak of *Andes orthohantavirus* (Andes virus, ANDV) aboard the *MV Hondius* has thus far resulted in 13 confirmed cases, including three fatalities, and has rapidly evolved into an international health crisis. ANDV is a zoonotic New World hantavirus whose main reservoir is the long-tailed colilargo, and that causes severe hantavirus cardiopulmonary syndrome (HCPS) in humans [1,2]. Transmission to humans usually occurs through contact with aerosols from secretions or excretions of infected rodents. Although several seasonal outbreaks occur regularly, mainly in Chile and Argentina, transmission chains are usually short-lived and involve limited person-to-person transmission [3,4]. Nevertheless, the recent outbreak is thought to have involved up to three generation transmission chains. Sequenced isolates from the *MV Hondius* outbreak harbour several nonsynonymous mutations throughout their genomes compared to reference strains and isolates from previous outbreaks, while exhibiting little genetic divergence within the 2026 outbreak cluster [5,6]. Such nonsynonymous mutations include two changes in the RdRp, one change in the N protein, and six changes affecting the glycoprotein complex (GPC) (Supplementary Table 1). Whether these sequence polymorphisms enhance ANDV capacity to infect human cells remains unknown.

Entry of hantaviruses into host cells is mediated by GPC, encoded in the M segment. We sought to assess the capacity of the GPC from an *MV Hondius* isolate (SWITZ/2026) to mediate entry into human cells and to compare its entry efficiency with those of a reference human strain (CHI-7913) and an isolate from a previous human outbreak in Argentina in 2018 (Epuyen/2018) (Figure 1(A)). Relative to CHI-7913, the Epuyen/2018 and SWITZ/2026 isolates each harbour six nonsynonymous amino acid substitutions in their GPC, both sharing only one substitution (I11 V, Figure 1(B)). To focus solely on viral entry, we generated single-cycle vesicular stomatitis virus (VSV) pseudotypes bearing the three ANDV GPCs. We then compared their infectivity across a panel of nine human cell lines, selected because they express known ANDV receptors [7,8] (PCDH1: 0.1-13.3 fragments per kilobase of transcript per million mapped reads [FPKMs], Integrin Subunit Alpha V: 0.9-6.2 FPKMs, Integrin Subunit Beta 3: 0.0-1.5 FPKMs) and because we previously demonstrated their susceptibility to the entry of other New World hantaviruses [9]. Additionally, we included Human Umbilical Vein Endothelial Cells (HUVECs) as a relevant primary endothelial cell model for ANDV infection. Viral titres spanned two orders of magnitude across all nine cell lines, with Huh-7 and ADR-RES cells exhibiting the highest and lowest susceptibility, respectively (Figure 1(C)). Titres of SWITZ/2026-GPC pseudotypes showed a strong positive correlation with those of CHI-7913 and Epuyen/2018 (r = 0.99 in both cases). Simple linear regression yielded slopes of 1.00 ± 0.04 (95% CI) and 0.95 ± 0.03 for CHI-7913 and Epuyen/2018, respectively, while estimates for the Y-intercept were 0.04 ± 0.25 (CHI-7913) and 0.38 ± 0.16 (Epuyen/2018). Taken together, these results indicate that the infectivity of SWITZ/2026 pseudotypes does not differ significantly from that of either CHI-7913 or Epuyen/2018 pseudotypes. Western blot analysis confirmed comparable incorporation of all three GPCs into VSV particles (Figure 1(D)). Finally, to rule out any potential confounding effects associated with the VSV backbone, we generated lentiviral pseudotypes bearing each ANDV GPC and similarly titrated them in human cell lines. Titres of SWITZ/2026 lentiviral pseudotypes did not differ significantly from those of CHI-7913 or Epuyen/2018 in any of the cell lines tested, further corroborating the findings described above (Figure 1(E)).

**Figure 1.**
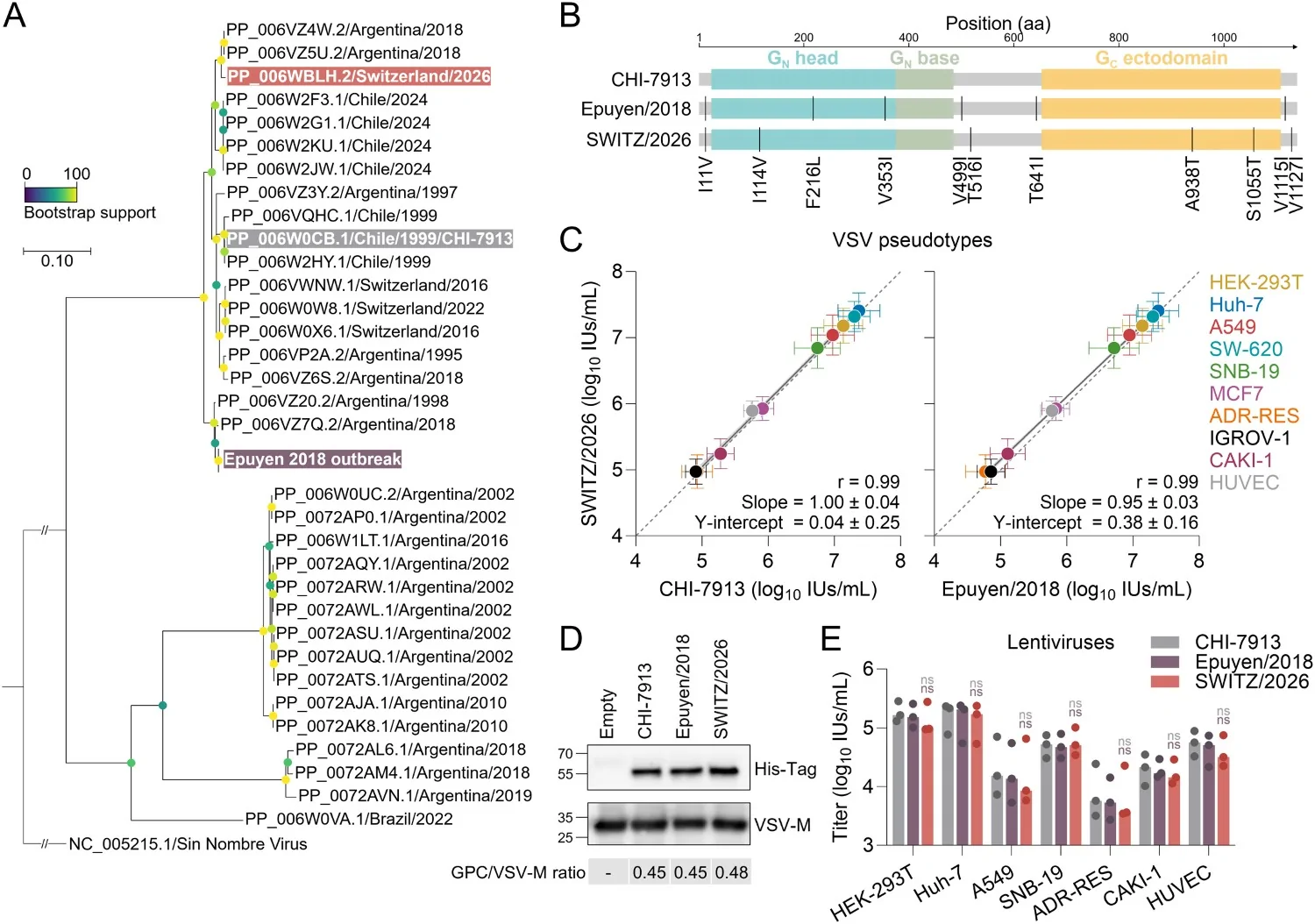
**Andes virus surface glycoproteins associated with different outbreaks exhibit similar entry into human cells.** (A) Phylogenetic analysis of M segments of Andes virus strains isolated in humans. Sixty nucleotide sequences corresponding to >90% complete M segments of ANDV isolates sampled from humans were retrieved from Pathoplexus and aligned with MAFFT. Phylogenetic reconstruction was performed using the TIM2 + F + G4 substitution model. Accession numbers are indicated at branch tips for each sequence. Sin Nombre virus was used as outgroup. Scale bar: 0.10 changes per site. (B) Schematics of surface glycoproteins from CHI-7913, Epuyen/2018 and SWITZ/2026 isolates. Non-synonymous changes compared to the CHI-7913 sequence are depicted by black lines, and specific amino acid changes are indicated below. G_C_ ectodomain and G_N_ head and base domains are indicated with coloured boxes. (C) VSV pseudotypes were titrated on the indicated cell lines. Data show the correlation between viral titres of SWITZ/2026 strain and CHI-7913 (left) or Epuyen/2018 (right), in infectious units per volume unit (IU/mL). Each dot corresponds to the average of n = 3 independent pseudotype production and titration experiments. Error bars show the SEM. The Pearson correlation r and best-fit values ± 95% confidence intervals for a simple linear regression are shown. (D) Incorporation of ANDV surface glycoproteins in VSV particles was assessed via Western Blot and quantified as the GPC/VSV-M ratio. (E) Lentiviral particles bearing each ANDV glycoprotein were titrated on the indicated cell lines. Bars are set at the mean. Each dot corresponds to an independent pseudotype production and titration experiment (n = 3). Statistical significance was assessed on log-transformed titres with a 2-way ANOVA with repeated measures considering each independent production and infection block as the matching factor and Tukey’s post-hoc test for multiple comparisons.

Collectively, our results suggest that the GPC of the ANDV strain isolated aboard the *MV Hondius* mediates entry into human cells as efficiently as that of previous isolates, arguing against enhanced viral entry as the main determinant of the outbreak. The observed transmission dynamics may instead reflect the confined, high-contact environment aboard the cruise ship, similar to what was reported during the Epuyén outbreak [10]. However, contributions from genetic variation in viral segments other than M, potentially affecting genome replication or other aspects of viral biology such as immune escape, tissue tropism, or viral shedding, cannot be excluded.

## Supplementary Material

SupplementaryTable1.xlsx
